# Variation of extrachromosomal circular DNA in cancer cell lines

**DOI:** 10.1016/j.csbj.2023.08.027

**Published:** 2023-08-28

**Authors:** Carl Rung dos Santos, Lasse Bøllehuus Hansen, Monica Rojas-Triana, Astrid Zedlitz Johansen, Mirna Perez-Moreno, Birgitte Regenberg

**Affiliations:** a Ecology and Evolution, Department of Biology, University of Copenhagen, Denmark; b Department of Oncology, Copenhagen University Hospital, Herlev and Gentofte, DK-2730 Herlev, Denmark; c Cell biology and Physiology, Department of Biology, University of Copenhagen, Denmark

**Keywords:** Cancer cell lines, Reproducibility, EccDNA, EcDNA, Double minute, CNV, non-mendelian

## Abstract

The presence of oncogene carrying eccDNAs is strongly associated with carcinogenesis and poor patient survival. Tumour biopsies and *in vitro* cancer cell lines are frequently utilized as models to investigate the role of eccDNA in cancer. However, eccDNAs are often lost during the *in vitro* growth of cancer cell lines, questioning the reproducibility of studies utilizing cancer cell line models. Here, we conducted a comprehensive analysis of eccDNA variability in seven cancer cell lines (MCA3D, PDV, HaCa4, CarC, MIA-PaCa-2, AsPC-1, and PC-3). We compared the content of unique eccDNAs between triplicates of each cell line and found that the number of unique eccDNA is specific to each cell line, while the eccDNA sequence content varied greatly among triplicates (∼ 0–1% eccDNA coordinate commonality). In the PC-3 cell line, we found that the large eccDNA (ecDNA) with *MYC* is present in high-copy number in an NCI cell line isolate but not present in ATCC isolates. Together, these results reveal that the sequence content of eccDNA is highly variable in cancer cell lines. This highlights the importance of testing cancer cell lines before use, and to enrich for subclones in cell lines with the desired eccDNA to get relatively pure population for studying the role of eccDNA in cancer.

## Highlights

• The eccDNA profile of cancer cell lines vary among triplicates.
• The number of unique eccDNA is specific to each cancer cell line.
• The NCI PC-3 isolate harbor *MYC* eccDNAs, while the ATCC isolate does not.
• It is important to test cell lines for presence/absence of eccDNA of interest.

## Graphical Abstract

**Figure:**
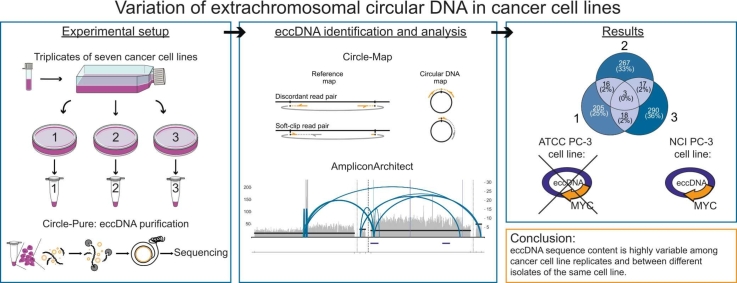

## 1 Introduction

Extrachromosomal circular DNAs (eccDNAs) are acentromeric circular DNA structures found in all eukaryotes and tissues tested so far [1], [2], [3], [4]. eccDNAs originate from chromosomal DNA and the size can vary from a few hundred basepairs to megabases [5], [6]. As such, eccDNAs are often subcategorized based on their size as either microDNA (<10^4^ base pairs) or ecDNA (10^4^-10^7^ base pairs) [4]. eccDNAs can carry full-length protein coding genes that, when expressed, can provide phenotypic advantages to host cells and allow them to adapt to unfavorable growth conditions [3]. This has been demonstrated in plants and in the single-celled eukaryotic model organism, *Saccharomyces cerevisiae*, where eccDNAs were shown to play a role in adaptation and survival [7], [8].

In cancer, oncogene-carrying eccDNAs can be a source of oncogene amplification across multiple cancer types and correlates with poor patient survival [4], [6], [9]. For example, *MYCN* has been found to be highly amplified via eccDNA in neuroblastoma [10], [11], *EGFR* in glioblastoma [10], [11], [12], and *ERBB2* in esophageal cancers [13]. The amplification of oncogenes is thought to provide a selective advantage to cancer cells over healthy cells by stimulating their growth and proliferation, fostering tumour development and chemotherapeutic resistance [10], [14]. The acentromeric nature of eccDNAs allows for unequal segregation during mitosis, in accordance with a Gaussian distribution, leading to the rapid generation of genetic heterogeneity in a dividing cell population [10], [15], [16]. As a result, there is a significant variation in the number of oncogene carrying eccDNAs in individual cancer cells, even in homogeneously defined cancer cell lines [6], [15]. Therefore, eccDNA has been investigated as a drug target, using cancer cell line models that recurrently harbour oncogene carrying eccDNAs, such as the PC-3 prostate cancer cell line, reported to harbour eccDNAs carrying the *MYC* gene resulting in its amplification [6], [10], [17], [18].

However, loss and variations in eccDNA content among cancer cell lines poses a challenge to reproducibility in research studies. As such, we aimed to address a key question: To what extent are cancer cell lines affected by variations in eccDNA content? The presence of an eccDNA in an eukaryotic cell is expected to be determined by five factors [3]: I) eccDNA formation rate, II) replication during mitosis, III) loss or elimination rate, IV) segregation during mitosis and, V) selective growth advantages provided to the host cells. Studies comparing eccDNA content in patient-derived cancer cell lines and matched tumours suggest that eccDNAs are often lost during prolonged *in vitro* growth of three weeks or more [19]. Earlier efforts at establishing stable eccDNA harbouring cancer cell lines by *de novo* cutting and ligating a chromosomal region together to form eccDNA, have failed because the cell lines gradually lose eccDNAs [20]. When cancer cell lines are propagated in culture, some eccDNAs tend to reintegrate into the chromosomes instead of staying extrachromosomal [21]. Finally, cancer cell lines are genetically unstable [22], and DNA replication stress has been shown to promote both the formation and loss of eccDNA [23], [24]. Therefore, we hypothesize that cancer cell lines exhibit a high turnover rate of eccDNA, leading to significant variations in eccDNA content.

We tested the hypothesis using two approaches: First, eccDNA was purified and sequenced from triplicates of seven cancer cell lines and identified using the Circle-Map pipeline [25]. This was done to assess inter-triplicate variations in the content of all unique eccDNAs in a cancer cell line population. Second, we applied the AmpliconArchitect pipeline [26] to assess variations in the presence of high-copy number eccDNAs, which are likely to be maintained in a cancer cell line population due to the selective advantage these eccDNAs provide to host cells. We find substantial inter-triplicate variations in the content of eccDNA and cell line isolate specific variations in high copy-number eccDNAs. These variations could have implications for the reproducibility and reliability of studies that use cancer cell lines to study the role of eccDNAs in cancer.

## 2 Materials and methods

### 2.1 Cell culture

Mouse cancer cell lines MCA3D, PDV, HaCa4, and CarC [27], [28], [29] were kind gifts of Dr. Amparo Cano (Instituto de Investigaciones Biomédicas Alberto Sols, Madrid, Spain). Human cancer cell lines MIA-PaCa-2 [30], AsPC-1 [31], and PC-3 [32] were supplied by Herlev Hospital and purchased from ATCC. The PC-3 cell line was authenticated by IDEXX BioAnalytics and confirmed to be the PC-3 cell line available at ATCC. Cells were cultured from a frozen stock (−80°C) until confluence was reached. Then, ∼300.000 cells were transferred to new petri dishes in triplicates and cultured until confluence was reached. Finally, ∼10^6^ cells were pelleted. Triplicates were collected on the same day. PC-3 and AsPC-1 cells were cultured in RPMI 1640, GlutaMAX, HEPES (cat.no. 72400021, Gibco), supplemented with 10% fetal bovine serum (FBS) (cat.no. 10500064, Gibco), and 1% penicillin/streptomycin (P/S) (cat.no. 15140122, Gibco). MCA3D, PDV, and HaCa4 cells were cultured in Ham’s F-12 Nutrient Mixture (cat.no. 11765054, Gibco), supplemented with 10% FBS, and 1% P/S. CarC and MIA-PaCa-2 cells were cultured in DMEM, GlutaMAX (cat.no. 11594446, Gibco), supplemented with 10% FBS, and 1% P/S. DPBS (cat.no. 14190169, Gibco) was used to wash adherent cells prior to detachment with trypsin-EDTA (0.05%) (cat.no. 25300054, Gibco). All cells were cultured at 37 °C with 5% CO_2_.

### 2.2 Circle-Pure eccDNA extraction, purification and amplification

eccDNA was extracted and purified using Circle-Pure (cat.no. 1001–24, CARE-DNA), that is based on the Circle-Seq method [33]. **1)** A cell pellet of ∼10^6^ cells was resuspended in 100 µl DNase/RNase free H_2_O (cat.no. 11538646, Invitrogen). Then, 750 µl α-buffer (CARE-DNA), 20 µl RNase A (cat.no. 19101, Qiagen), and 50 µl Proteinase K (cat.no. EO0492, Thermo Scientific) was added and the suspension was incubated for 30 min at room temperature (25 °C). Thereafter, 552 µl AMPure XP beads (0.6X current volume) (cat.no. A63881, Beckman Coulter) and 1400 µl β-buffer (CARE-DNA) was mixed with the sample by pipetting. Beads were aggregated on a magnetic rack and washed twice in 3000 µl 80% ethanol. Then, beads were resuspended in 55 µl Elution Buffer (CARE-DNA), incubated at 50 °C for 5 min, and beads were aggregated to elute the DNA suspension. This was repeated once more to reach a final volume of 110 µl total DNA. **2)** To remove linear DNA, 40 µg total DNA was mixed with 10 µl NEBuffer 4 (10X), 3 µl Exonuclease V (10,000 U/ml) (cat.no. M0345L, NEB), 10 µl ATP (10 mM), and nuclease-free H_2_O to reach a final volume of 100 µl. This was incubated for 3 days at 37 °C. Every 24 h additional 1.4 µl NEBuffer 4 (10X), 3 µl Exonuclease V (10,000 U/ml), and 10 µl ATP were added to the reaction. The reaction was inactivated by incubation at 70 °C for 30 min. Thereafter, mtDNA was linearized using CRISPR-Cas9 with sgRNAs targeting two mtDNA positions [34]. To remove linearized mtDNA and residual linear DNA, another 4 days of exonuclease treatment were performed. The pre-mtDNA linearization exonuclease treatment is performed to remove linear DNA to limit the chance of linear DNA acting as an off-target for the CRISPR-Cas9 sgRNAs. mtDNA and linear DNA removal was confirmed by PCR. Purified eccDNA was amplified using TruePrime Phi29 rolling circle amplification (cat.no. 390100, 4basebio) for 48 h.

### 2.3 mtDNA linearization

Targeted removal of mitochondrial DNA from eccDNA was performed according to previously published protocol [34] using the Cas9 Nuclease, *S. pyogenes* (cat.no. M0386M, NEB). sgRNAs were synthesized using the EnGen sgRNA Synthesis Kit, *S. pyogenes* (cat.no. E3322S, NEB) according to the manufacturer’s protocol. Mouse-sgRNA1: GTAGCATGAACGGCTAAACGA. Mouse-sgRNA2: GGCCTGATAATAGTGACGCT. Human-sgRNA1: GGCTTGGATTAGCGTTTAGA. Human-sgRNA2: GCGTAGGGGCCTACAACGTTG.

### 2.4 Pulsed-field gel electrophoresis

Pulsed-field gel electrophoresis (PFGE) was carried out using the CHEF-DR II Pulsed Field Electrophoresis System (Bio-Rad). A 1% agarose gel was cast using Pulsed Field Certified Agarose (cat.no. 1620137, Bio-Rad) in fresh 0.5X TBE buffer. A CHEF DNA Size Marker, 0.2–2.2 Mb, *S. cerevisiae* Ladder (cat.no. 1703605, Bio-Rad) agarose plug was loaded in the first lane as a size reference. One µg total DNA with a volume of 40 µl was loaded in each well. The PFGE ran for 40 h at 14 °C, at 5 V/cm, initial SW: 47 s, and final SW: 170 s. The gel was post-stained in a 3X GelRed solution for 30 min.

### 2.5 PCR, gel electrophoresis and Sanger sequencing

Thermo Scientific DreamTaq PCR Master Mix (2X) (K1071) was used for all PCR reactions. PCR products ran on a 0.7–2% agarose gel in 1X Bionic buffer (Sigma-Aldrich) to verify that the PCR reaction progressed as expected. The percentage of agarose was determined by how large a PCR product was expected. Monarch® DNA Gel Extraction Kit Protocol (NEB #T1020) was used to extract DNA from a gel slice and purify the PCR product for Sanger sequencing. Sanger sequencing was performed by Eurofins Genomics.

### 2.6 Metaphase chromosome fixation

PC-3 cells were cultured in 150 mm round plates with RPMI 1640, GlutaMAX, HEPES supplemented with 10% FBS and 1% P/S till 70% confluency was reached. Cells were treated with KaryoMAX (cat.no. 15212012, Gibco) at a final concentration of 0.01 μg ml^−1^ for 3.5 h and collected using Trypsin-EDTA 0.25%. After collection, the cells were subjected to a hypotonic treatment in pre-warmed (37 °C) 0.075 M KCl for 30 min at 37 °C. The osmotically swollen cells were fixed by gently resuspending in increasing volumes (3 drops, 0.5 ml, and 3 ml) of Carnoy’s fixative solution (1:3 glacial acetic acid:methanol). For metaphase chromosomes spreads, the fixed cells were dropped onto ice-cold humidified glass slides and store at − 20 °C until FISH analysis was performed.

### 2.7 Fluorescense in situ hybridization (FISH)

Metaphase chromosome spreads were dehydrated in an ethanol series with increasing concentration (70%, 85%, and 100%). Once the slides were dry, 15 µl of probe hybridization mixture were added to each slide, covered with a coverslip, and sealed with rubber cement. The hybridization mixture consisted of FISH hybridization buffer (cat.no. G9400A, Agilent) and the oligonucleotide-based FISH probe SureFISH 8q24.21 MYC 294 kb with Cy3 (cat.no. G110365R-8, Agilent). Co-denaturation of the probe and chromosomal DNA was carried out at 78 °C for 5 min and the hybridization reaction was incubated for 16 h in a humidified chamber at 37 °C. Slides were subsequently washed in 0.4x saline-sodium citrate (SCC) (pH 7.0) for 2 min at 50 °C, followed by a second wash in 2x SCC 0.05% Tween-20. The FISH slides were mounted using ProLong Gold antifade mounting reagent (cat.no. P36930, Invitrogen) supplemented with DAPI at a final concentration of 2.5 μg ml^−1^. Images were captured with a Leica SP5 X confocal microscope at a magnification of 63x increased with 2–4x zoom. Images were edited using LAS X Office (Leica Microsystems).

### 2.8 Whole-genome sequencing

DNA sequencing libraries were prepared using NEBNext Multiplex Oligos for Illumina (cat.no. E6440S, NEB) and NEBNext Ultra II DNA Library Prep Kit for Illumina (cat.no. E7645L, NEB). 500 ng amplified eccDNA/total DNA was fragmented by sonication to obtain fragment lengths of ∼ 400 bp. AMPure XP beads (cat.no. A63881, Beckman Coulter) were used for size-selection of adaptor-ligated DNA fragments to reach ∼ 400–600 bp. DNA libraries were pooled and paired-end (150 bp) sequenced on an Illumina NovaSeq 6000. Sequence reads were trimmed for adaptor content with BBduk (version 38.90). Reads were quality checked with FASTQC (version 0.11.9), and aligned against the mouse reference genome mm10 (GCA_000001635.2) or human reference GRCh38 (GCA_000001405.15) with BWA MEM (version 0.7.17). Genomic features were annotated with Bedtools intersect (version 2.30.0). The gencode.vM10.annotation.gtf.gz file was used to annotate genomic features on eccDNA coordinates in mouse cancer cell lines. The gencode.v42.annotation.gtf.gz file was used to annotate genomic features on eccDNA coordinates in human cancer cell lines. SAMtools (version 1.9) was used to sort.bam files and calculate sequencing read depth. Picard-tools (version 2.26.10) were used to mark duplicate reads and perform downsampling. CNVkit (version 0.9.9) was used to find copy-number variants based on sequencing read depth [35].

### 2.9 Data acquisition

WGS data on PC-3 from Seim et al. was acquired from bioproject PRJNA361315 run SRR5196724. WGS data on PC-3 from Turner et al. was acquired from bioproject PRJNA338012 run SRR4009277. sratoolkit (version 2.11.3) https://github.com/ncbi/sra-tools was used for downloading the data.

### 2.10 Identification of eccDNA with Circle-map

eccDNA sequencing data from samples purified with Circle-Pure was analyzed using the Circle-Map Realign [25] bioinformatics pipeline to map eccDNA coordinate as detailed at: https://github.com/iprada/Circle-Map.

### 2.11 Identification of eccDNA with AmpliconArchitect

WGS data from total DNA of PC-3 extracted with Circle-Pure, from Seim et al., and Turner et al. was analyzed with AmpliconArchitect to find focal amplifications in the form of eccDNA [26]. AmpliconArchitect analysis was performed following the pipeline with default setting as detailed at: https://github.com/virajbdeshpande/AmpliconArchitect. AmpliconClassifier (version 0.4.12) [13] https://github.com/jluebeck/AmpliconClassifier was used to determine whether amplicons were classified as eccDNA. CycleViz https://github.com/jluebeck/CycleViz was used to visualize amplicons classified as eccDNA identified by AmpliconArchitect.

## 3 Results

### 3.1 The number of unique eccDNA is cancer cell line specific

**Fig. 1:**
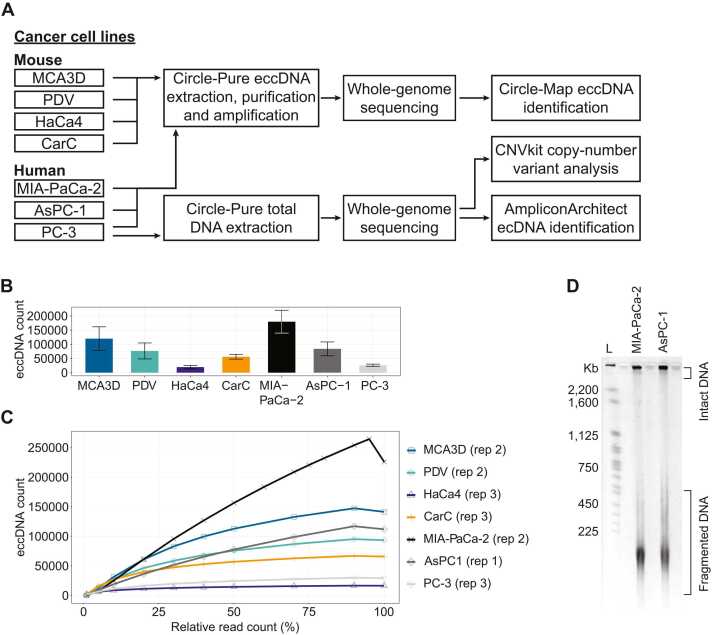
Workflow and summary of eccDNA extracted with Circle-Pure identified with Circle-Map. A) Schematic representation of the study workflow. B) Mean number of unique eccDNA (n = 3) identified by Circle-Map. The presence of all eccDNAs reported in the mouse cancer cell lines is supported by at least 1 soft-clipped read, 1 discordant read-pair, and at least 90% read coverage to be considered valid. The presence of all eccDNAs reported in human cancer cell lines is supported by at least 4 soft-clipped reads, 4 discordant read-pairs, and at least 99% read coverage to be considered valid. C) Saturation plot of relative read count percentage against the eccDNA count of the cell line replicates with highest eccDNA count/million mapped reads. D) A PFGE gel of total DNA extracted from MIA-PaCa-2 (rep 3) and AsPC-1 (rep 3) with Circle-Pure. L = CHEF DNA Size Marker, 0.2–2.2 Mb, *S. cerevisiae* Ladder.

To investigate the variation of eccDNA in cancer cell lines, we cultured seven cancer cell lines of different origin in triplicates and collected cell pellets consisting of ∼10^6^ cells. We extracted, purified, and amplified eccDNA from the cell pellets using the Circle-Pure method. Linear chromosomal DNA removal and mtDNA reduction was verified by PCR (Supplementary Fig. 1a-b). The amplified eccDNA was then sequenced and the bioinformatics pipeline Circle-Map [25] was applied to identify every unique eccDNA present in each cancer cell line (Fig. 1a). The presence of each eccDNA identified in the mouse cancer lines (MCA3D, PDV, HaCa4, and CarC) was required to be supported by at least 1 soft-clipped read, 1 discordant read pair, and have at least 90% read coverage to be considered valid (Supplementary Fig. 2a). Similarly, the presence of each eccDNA identified in the human cancer lines (MIA-PaCa-2, AsPC-1, and PC-3) was required to be supported by at least 4 soft-clipped reads, 4 discordant read pairs, and have at least 99% read coverage to be considered valid (Supplementary Fig. 2b). The soft-clipped read and discordant read thresholds are different because human and mouse cell lines were sequenced at different depths (Table 1). We found that the number of unique eccDNA is specific to each cancer cell line, independent of sequencing depth (Fig. 1b, Table 1). We downsampled the sequencing reads of the cell line replicates with the highest number of eccDNA/million mapped reads. Downsampling indicated that sufficient read depth was achieved to capture most of the unique eccDNAs from an extract of ∼10^6^ cells of all cell lines investigated except for MIA-PaCa-2 (Fig. 1c). The kink seen in the downsampling curve from 90% to 100% relative read count (%), is a result of chromosomal regions covered by reads that support smaller eccDNAs being merged by Circle-Map into a single larger eccDNA. It has previously been postulated that only small eccDNA (up to tens of kilobases) can be extracted using in-solution magnetic-bead-based DNA extraction methods [36]. However, we find eccDNAs extracted with Circle-Pure larger than 100 kilobases in the mouse and human cancer cell lines (Supplementary Fig. 2c). Furthermore, we demonstrate that with the Circle-Pure method, intact ultrahigh-molecular weight (UHMW) total DNA can be extracted (Fig. 1d). Altogether, our findings demonstrate that the number of unique eccDNA is specific to each cancer cell line and that eccDNAs of all sizes can be extracted from a mammalian cell pellet using the Circle-Pure method.

**Table 1:** Summary of sequencing quality and eccDNAs identified and in the mouse and human cancer cell lines.

Sample	Mapped reads	Percentage of mapping	Average read depth/base	No. of unique eccDNA^a^	No. of unique eccDNA/million mapped reads
Mouse cancer cell lines:					
MCA3D (1)	201443167	99.65%	10.156	146611	728
MCA3D (2)	138051319	99.68%	7.04703	141355	1024
MCA3D (3)	128065374	99.54%	6.56393	71918	562
PDV (1)	100439887	99.21%	5.14933	44072	439
PDV (2)	129758437	99.60%	6.67405	93450	720
PDV (3)	150194197	99.40%	7.71015	92339	615
HaCa4 (1)	159188662	98.13%	8.1033	26547	167
HaCa4 (2)	120115453	97.80%	6.10369	15944	133
HaCa4 (3)	92840060	92.28%	4.70453	16431	177
CarC (1)	161397780	99.18%	8.27452	52226	324
CarC (2)	170075710	98.50%	8.67632	50420	296
CarC (3)	130045216	99.45%	6.58557	65782	506
Human cancer cell lines:					
AsPC-1 (1)	576510849	99.92%	26.881	111879	194
AsPC-1 (2)	495893974	99.90%	23.2378	67085	135
AsPC-1 (3)	599741512	99.90%	28.0021	72972	122
MIA-PaCa-2 (1)	514563258	99.92%	24.0371	150710	293
MIA-PaCa-2 (2)	569543644	99.91%	26.5841	225789	396
MIA-PaCa-2 (3)	442337822	99.92%	20.645	162924	368
PC-3 (1)	595013127	99.22%	26.6893	21861	37
PC-3 (2)	581145025	99.28%	25.4111	27661	48
PC-3 (3)	469289289	99.14%	20.7999	29300	62

a In mouse cancer cell lines, the presence of each eccDNA is supported by at least 1 discordant read, 1 soft-clipped read, and at least 90% read coverage to be considered valid. In human cancer cell lines, the presence of each eccDNA is supported by at least 4 discordant reads, 4 soft-clipped reads, and at least 99% read coverage to be considered valid.

### 3.2 eccDNA content vary substantially among cancer cell line triplicates

**Fig. 2:**
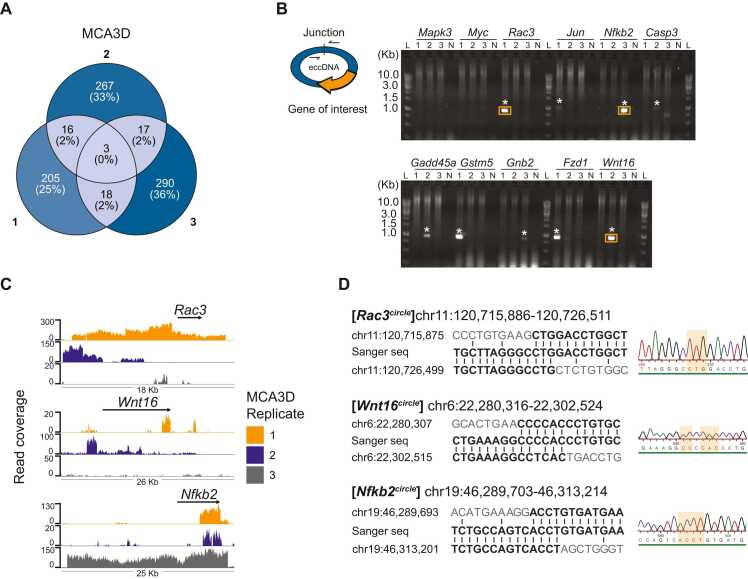
Inter-replicate eccDNA variation. A) Venn diagram of unique full-length protein-coding genes located on eccDNA in each replicate (designated 1, 2, and 3) of the MCA3D cell line. Full-length protein coding genes located on eccDNA found in three replicate: *Krtap10–4*, *Gm10840*, *Psmg3*. Of the total number of unique eccDNAs identified in the MCA3D cell line, between ∼ 0.2–0.5% carry a full-length protein coding gene. B) Outwards PCR spanning the junction site of 11 eccDNAs identified in MCA3D that carry a known oncogene. Amplified eccDNA was used as the template DNA for the PCR reaction. The expected band size ranged from 0.8 kb to 1.2 kb. L = GeneRuler 1Kb DNA ladder. N = negative control. Lane numbers refer to replicate numbers. Stars indicate correct size of PCR product and orange boxes refer to the gel slices that were extracted for Sanger sequencing. C) eccDNA read coverage plot of *Rac3*^*circle*^, *Wnt16*^*circle*^ and *Nfkb2*^*circle*^. D) Sanger sequencing results of the PCR bands highlighted in orange in B.

To determine the degree of eccDNA variation in cancer cell lines, we annotated genetic features on the eccDNA chromosomal coordinates and compared the triplicates of each cell line. Of the total number of full-length protein-coding genes located on eccDNA, between 0% and 2% reoccur in triplicates of each investigated cancer cell line (Fig. 2a, Supplementary Fig. 3). None of these recurring genes have been correlated with carcinogenesis, cell proliferation, or were related to growth (Fig. 2a, Supplementary Fig. 3). However, in the MCA3D cell line, multiple cancer-associated genes were found on eccDNAs in individual replicates. In replicate 1, individual eccDNAs carrying *Rac3*, *Jun*, *Gstm5*, and *Fzd1* were identified. In replicate 2, individual eccDNAs carrying *Casp3*, *Gadd45a*, and *Wnt16* were identified. In replicate 3, individual eccDNAs carrying *Mapk3*, *Myc*, *Nfkb2*, and *Gnb2* were identified. We verified the presence of nine of these by outwards PCR spanning the eccDNA junction site (Fig. 2b). Further, we Sanger sequenced the PCR product of the [*Rac3*^circle^], [*Wnt16*^*circle*^], and [*Nfkb2*^*circle*^] to confirm that the outwards PCR specifically amplified the eccDNA junction. This revealed that these eccDNAs were formed from regions of microhomology (Fig. 2c-d). MCA3D is an immortalized cell line that originates from mouse keratinocytes and exerts an epithelial morphology *in vitro*. MCA3D cells are non-tumorigenic when injected into immunocompromised mice. To have a non-genic measure of eccDNA variance, we examined how many unique eccDNAs the cancer cell line triplicates have in common based on their chromosomal coordinates + /- 200 basepairs in both start and end coordinate. Out of the total number of unique eccDNAs identified in the cancer cell lines, between 0.1% and 0.3% are found in two replicates of the same cell line. When comparing triplicates, the eccDNA commonality is almost negligible (Table 2). Taken together, these results demonstrate that the eccDNA content in individual cultures of the same cancer cell line population is highly variable.

**Table 2:** Number of unique eccDNA with same chromosomal coordinates allowing 200 basepairs + /- overlap in both start and end eccDNA coordinate.

Cell line	Replicate 1 + 2	Replicate 1 + 3	Replicate 2 + 3	Replicate 1 + 2 + 3	eccDNA coordinate commonality in 2 replicates^a^	eccDNA coordinate commonality in 3 replicates^a^
Mouse cancer cell lines:						
MCA3D	641	279	294	4	0.3%	0.003%
PDV	99	133	225	1	0.2%	0.001%
HaCa4	10	4	9	0	0.1%	0.000%
CarC	81	72	100	2	0.2%	0.004%
Human cancer cell lines:						
AsPC-1	156	150	99	2	0.2%	0.002%
MIA-PaCa-2	407	332	532	1	0.2%	0.001%
PC-3	46	50	48	9	0.2%	0.034%

a Percent average of the total number of unique eccDNA/number of eccDNA that has the same chromosomal coordinates in either two or three replicates (+/- 200 basepairs).

### 3.3 The presence of *MYC* eccDNA in PC-3 cells is isolate specific

**Fig. 3:**
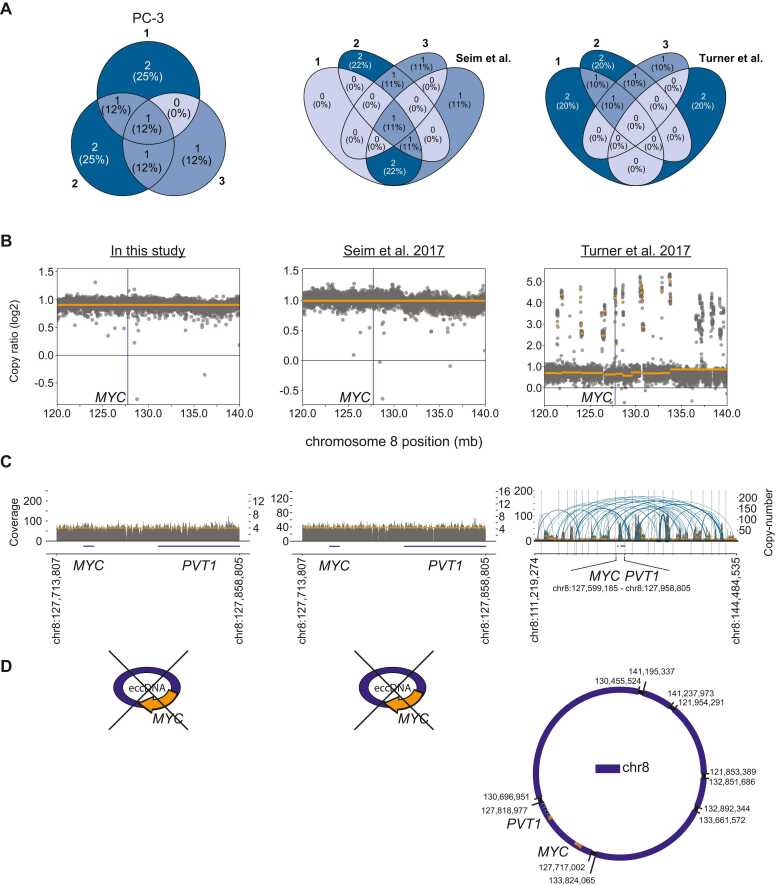
Copy-number variation and eccDNA identification in PC-3. A) Venn diagram of genes located on amplified eccDNAs (copy number > 5) in PC-3 cells from this study (left), comparison with Seim et al. (middle), and comparison with Turner et al. (Right). B) CNVkit scatterplot of chr8:120–140 Mb of the PC-3 cell line isolate from this study (left), Seim et al. (middle), and Turner et al. (Right). The y-axis describes the log(2) copy ratio reported by CNVkit and represents the deviation in copy number of each genomic segment in the sample relative to the expected copy number based on the reference genome [35]. The vertical purple line represents the location of the *MYC* gene (∼127 Mb). C) AmpliconArchitect output of the amplicon that included the *MYC* gene in the PC-3 cell line isolate from this study (left), Seim et al. (Middle), and Turner et al. (Right). Read coverage is represented as grey coverage bars and absolute copy-number is represented as horizontal orange lines. The location of the *MYC* and *PVT1* gene is shown as purple lines underneath the graphs. D) No *MYC*-carrying eccDNAs were identified in the PC-3 cell line isolate from this study (left) or with data from Seim et al. (middle). With the Turner et al. WGS data, *MYC* and *PVT1* carrying eccDNAs were identified by AmpliconClassifier and visualized with CycleViz (right).

**Fig. 4:**
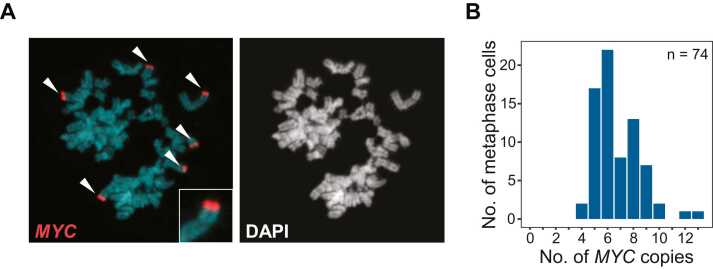
*MYC* gene copy number in PC-3 cells by FISH analysis. A) Representative FISH metaphase chromosomes in PC-3 cells with *MYC* (red) and stained with DAPI (cyan) for DNA detection. B) Number of *MYC* copies detected in 74 PC-3 metaphase chromosome spreads. Median number of *MYC* copies per cell = 6.

To assess the variability in the presence of maintained eccDNAs in cancer cell lines, we whole-genome sequenced total DNA from triplicates of the PC-3 cell line (Fig. 1a). Prior studies have reported *MYC* amplification through eccDNA in this cell line [6], [10], [17], [18]. We investigated copy-number variations (CNV) and applied the AmpliconArchitect [26] (AA) pipeline to identify eccDNA focal amplifications in our own PC-3 triplicates and two publicly available PC-3 WGS datasets from Seim et al. [37] and Turner et al. [6]. A total of twelve amplified eccDNAs (copy number > 5) that carried one or more genes were identified in our PC-3 triplicates. Of those, one was present in all three replicates and two were present in two replicates (Fig. 3a, left). None of these were identified using the Circle-pure approach (Fig. 1b). In the PC-3 isolate from Seim et al., 4/5 amplified eccDNAs identified were also present in at least one of our PC-3 replicates (Fig. 3a, middle). In the PC-3 isolate from Turner et al., none of the amplified eccDNAs identified, were also present in our replicates (Fig. 3a, right). However, we found *MYC* carrying eccDNAs in the Turner et al. PC-3 isolate which were not identified in our own nor the Seim et al. isolate (Fig. 3b-d, Supplementary table 1, Supplementary Fig. 4a). We compared the global CNV profile of the five datasets, and found that the Turner et al. PC-3 isolate differs from ours and that from Seim et al. (Supplementary Fig. 4b). Both ours and the Seim et al. PC-3 isolate were purchased from the American Type Culture Collection (ATCC), whereas the isolate from Turner et al. was given as a gift by the National Cancer Institute (NCI). To verify the absence of *MYC* carrying eccDNAs in our PC-3 isolate, we employed fluorescence in situ hybridization (FISH) using probes targeting the *MYC* gene. We found that the vast majority of *MYC* signals overlapped with the main body of chromosomes rather than being outside the chromosomes, indicating that *MYC* carrying eccDNAs are either rare or not present in our PC-3 isolate (Fig. 4, Supplementary Fig. 5). Altogether, these results show isolate specific variations in the presence of eccDNA that are present in high copy number.

## 4 Discussion

Here we show that the eccDNA sequence content in cancer cell lines is highly variable and the number of unique eccDNA is specific to each cancer cell line investigated. These results suggest that the variability comes from a continuously high rate of eccDNA formation and loss, specific to each cell line [3]. In the MCA3D cell line we identified oncogene carrying eccDNAs but each were private to a single replicate. We argue that the eccDNAs identified are carrying oncogenes because of random chance rather than enrichment of cells that harbour oncogenic eccDNA as a result of selection. Our findings also revealed that *MYC* carrying eccDNAs in the PC-3 cell line is present in an NCI isolate and is not identified in the ATCC isolates investigated in this study. Previous studies have identified many copies of *MYC* carrying eccDNAs in PC-3 cells acquired from ATCC [17], [18]. Therefore, we suggest that *MYC* carrying eccDNAs in ours and Seim et al. PC-3 isolates were lost during the propagation of the cell line as result of unequal mitotic segregation. This is not surprising as previous studies analyzing metaphase spreads of cancer cell lines have found that cancer cell lines consist of a heterogeneous mixture of cells, that include cells that harbour oncogene-carrying eccDNAs and cells that do not [6]. As such, researchers can potentially end up with a culture that mainly consists of cells that do not harbour the oncogene carrying eccDNA of interest. Mischel and coworkers have shown that cancer cells propagated in a selective environment for an extended period of time accumulate eccDNAs that enable them to adapt to such an environment. When propagated in a non-selective environment the number of these eccDNAs diminishes over time and remain low [10]. This has also been observed in *S. cerevisiae* [8], [38]. Finally, loss of genetic driver alterations and large inter-laboratory genetic and transcriptional differences that result in deviations in drug response, have previously been observed in multiple cancer cell lines [22], [39]. Altogether, this suggests that eccDNAs expected to be maintained in a cancer cell line due to the selective growth advantage they provide to host cells, can be lost when cancer cell lines are propagated *in vitro*.

Understanding the role of eccDNAs in cancer is crucial for the development of effective cancer therapies. Therefore, cancer cell lines that consistently harbour the same oncogene-carrying eccDNA in many copies, as a result of the advantage it provides to the host, continue to be a valuable resource for studying the fundamental biology of eccDNAs in cancer. It is important to acknowledge that the eccDNA variability observed here is not an error, but rather a consequence of the inherent nature of eccDNA to undergo changes. Therefore, we advise testing cancer cell lines before use, and to enrich for subclones in cell lines with the desired eccDNA to get relatively pure population for studying the role of eccDNA in cancer.

## Funding

We had funding from the 10.13039/501100009708Novo Nordisk Foundation (NNF21OC0072023) to B.R., M.P.M., and C.R.d.S. European Union’s Horizon 2020 research and innovation action under the FET-Open Programme (899417-CIRCULAR VISION) to B.R. and A.Z.J. 10.13039/100012774Innovation Fund Denmark under the Grand Solutions programme (8088-00049B CARE DNA) to B.R., A.Z.J., and L.B.H.

## CRediT authorship contribution statement

C.R.d.S. and B.R. designed and conceived the project. C.R.d.S., M.R.T. and A.Z.J. performed the experiments. C.R.d.S. analyzed the data and prepared figures. C.R.d.S, B.R., L.B.H., and M.P.M. designed the experiments. B.R. and L.B.H. supervised the study. C.R.d.S. and B.R. wrote the manuscript, and all authors edited the manuscript. All authors have read and approved the final version.

## Declaration of Competing Interest

B.R. is cofounder of CARE-DNA. All other authors declare that they have no known competing financial interests or personal relationships that could have appeared to influence the work reported in this paper.

## Appendix A Supplementary material

**Supplementary figure 1. Linear and mtDNA removal confirmation.** A) PCR with primers that amplify a region of ∼1.5 Kb in the *COX5B* gene on chr2 in humans and chr1 in mice. T = Total DNA. E = Exonuclease treated DNA. L = GeneRuler 1Kb Plus DNA ladder. B) PCR with primers that amplify a region in the ChrM of ∼400 bp in mice and ∼200 bp in humans. T = Total DNA. E = CRISPR linearized mtDNA and exonuclease treated DNA for linear DNA removal. In leftmost gel L = GeneRuler 1Kb Plus DNA ladder, in rightmost gel L = GeneRuler 1Kb DNA ladder.

.

**Supplementary figure 2. Integrative Genome Viewer read mapping examples.** A) A 50 Kb window of the MCA3D (2).bam file loaded in Integrative Genome Viewer (IGV) to display where reads align in the reference genome. Green reads represent soft-clipped reads. Dark blue reads represent discordantly mapped reads. The underlying graph denotes the position of specific genomic features. B) a 100 Kb window of the PC-3 (2) bam file loaded in IGV. C) Violin plots of eccDNA size in the cancer cell lines investigated. The y-axis represents the log_10_ eccDNA size (bp) and the x-axis represents the cancer cell lines.

.

**Supplementary figure 3.** Venn diagrams of unique full-length protein-coding genes located on eccDNA in the triplicates (designated 1, 2, and 3) of all the cell lines investigated. Full-length protein coding genes located on eccDNA found in three replicate of PDV: *Olfr361*, *Ufsp1*. Full-length protein coding genes located on eccDNA found in three replicate of MIA-PaCa-2: *OR10J3*, *OR10A5*, *OR4L1*, *OR1E2*, *SCP2D1*, *KRTAP21–3*, *KRTAP19–8*, *H2BC10*, *ATP6V1G2*, *OR2A2*, *and ENSG00000287585*. Full-length protein coding genes located on eccDNA found in three replicate of AsPC-1: *TAF11L2*. Of the total number of eccDNAs identified in each cell line between ∼ 0.1–0.2% carry a full-length protein coding gene.

.

**Supplementary figure 4**. **Copy-number variations in PC-3.** A) CNVkit scatterplot of chr8:120–140 Mb of the PC-3 replicate 2 and 3 from this study. B) Copy-number variations across all chromosomes calculated using CNVkit from the PC-3 triplicate WGS data, Seim et al. WGS data and Turner et al. WGS data. We note that the differences in copy number reported for the Turner et al. PC-3 isolate may be skewed as sequencing depth was low.

.

**Supplementary figure 5. FISH analysis PC-3 cells.** A) FISH collection of full metaphase spreads with the *MYC* (red) specific probe and stained with DAPI for DNA detection (cyan). B) potential *MYC* carrying eccDNA candidates (eccDNA; red arrow) or unspecific signals (blue arrows).
