# Supplementary figures and images for: Variation of extrachromosomal circular DNA in cancer cell lines
Source: Comput Struct Biotechnol J. 2023 Aug 28;21:4207–14. doi: 10.1016/j.csbj.2023.08.027 (PMC10495552; doi:10.1016/j.csbj.2023.08.027)

**A**

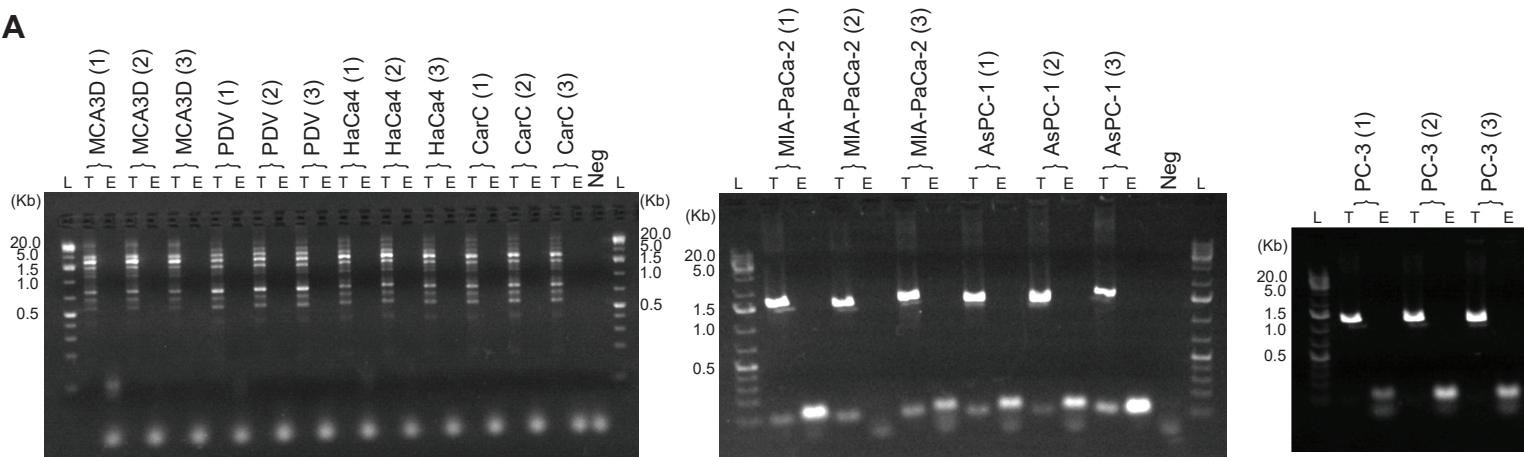

**B**

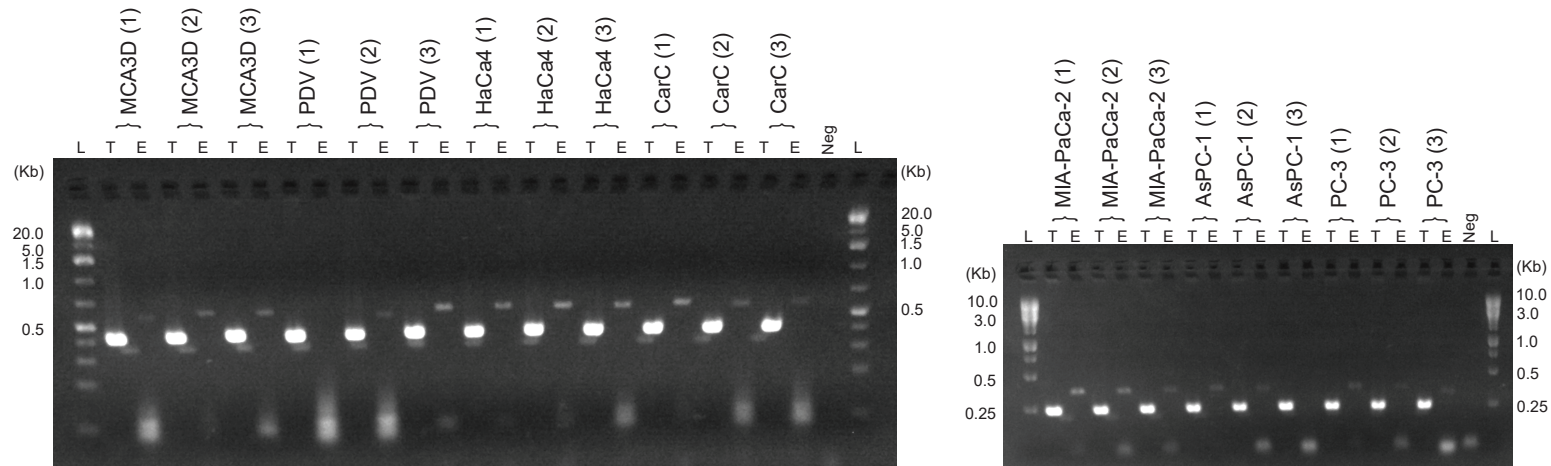

Supplement: Supplementary file 1 — Supplementary figure 1. Linear and mtDNA removal confirmation. A) PCR with primers that amplify a region of ∼1.5 Kb in the COX5B gene on chr2 in humans and chr1 in mice. T = Total DNA. E = Exonuclease treated DNA. L = GeneRuler 1Kb Plus DNA ladder. B) PCR with primers that amplify a region in the ChrM of ∼400 bp in mice and ∼200 bp in humans. T = Total DNA. E = CRISPR linearized mtDNA and exonuclease treated DNA for linear DNA removal. In leftmost gel L = GeneRuler 1Kb Plus DNA ladder, in rightmost gel L = GeneRuler 1Kb DNA ladder. [file mmc1.pdf]

**A**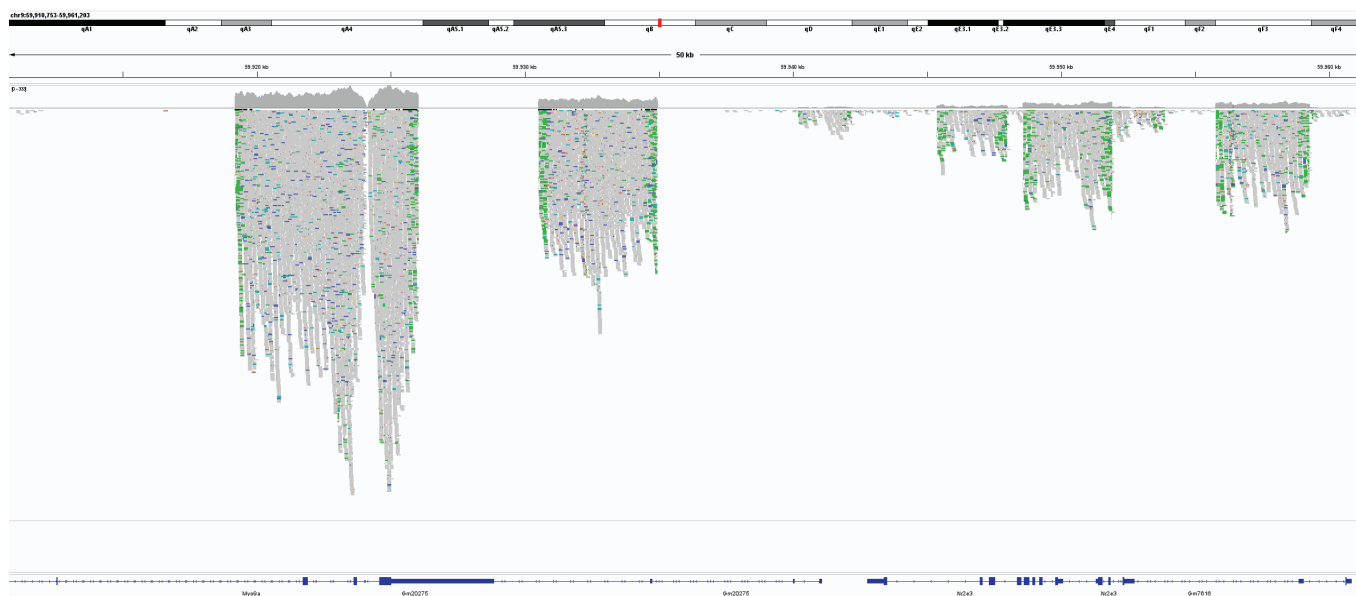**B**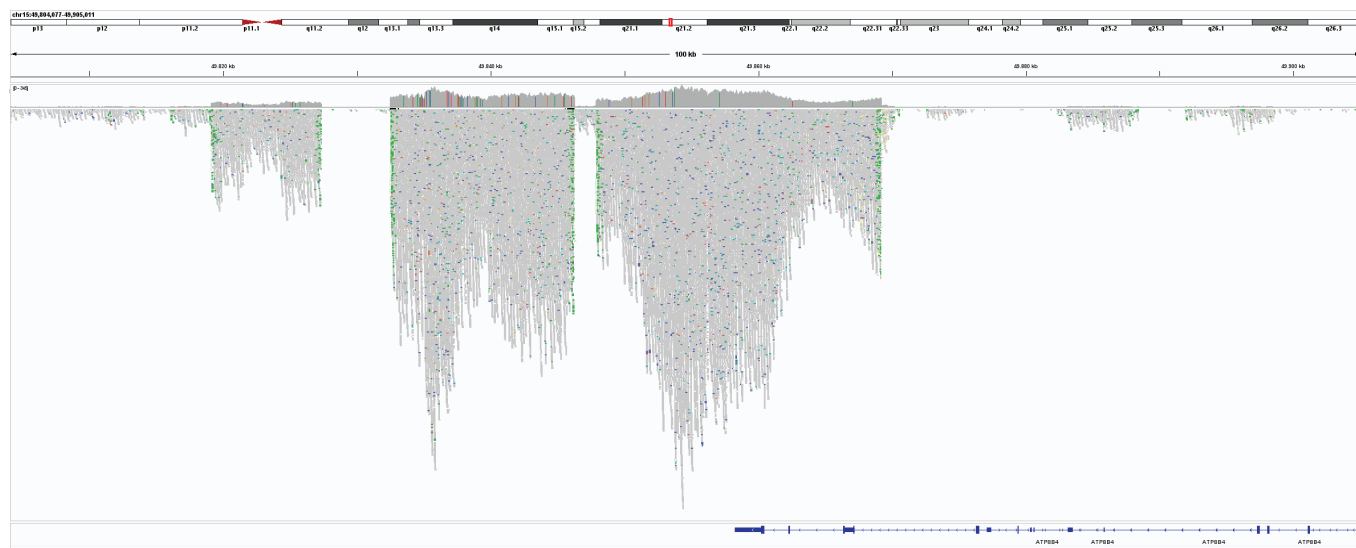**C**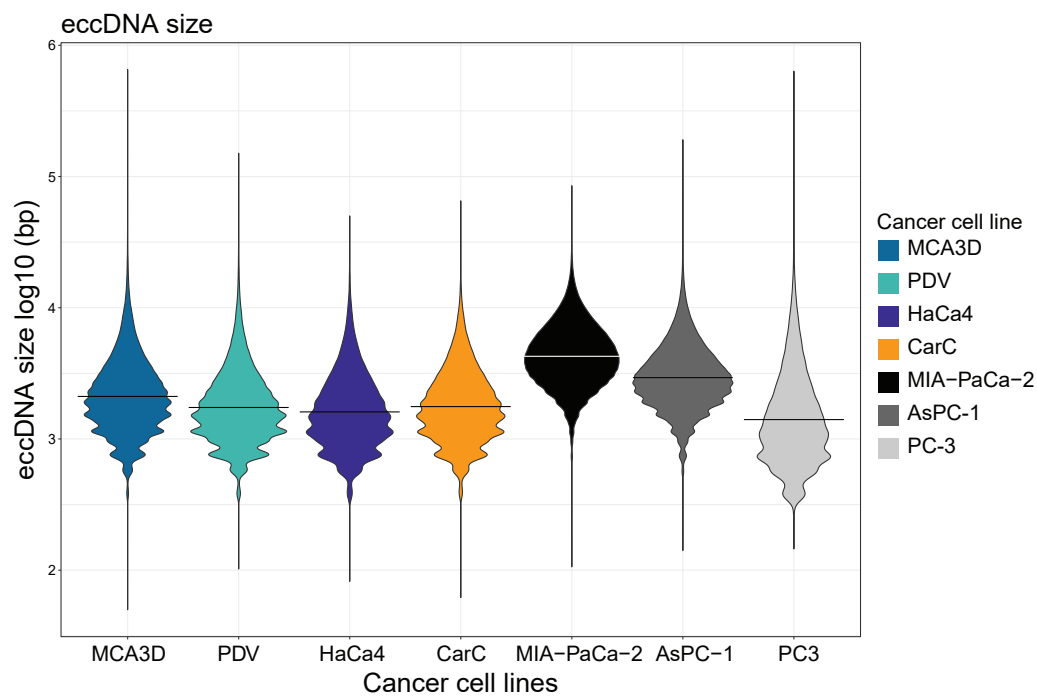

Supplement: Supplementary file 2 — Supplementary figure 2. Integrative Genome Viewer read mapping examples. A) A 50 Kb window of the MCA3D (2).bam file loaded in Integrative Genome Viewer (IGV) to display where reads align in the reference genome. Green reads represent soft-clipped reads. Dark blue reads represent discordantly mapped reads. The underlying graph denotes the position of specific genomic features. B) a 100 Kb window of the PC-3 (2) bam file loaded in IGV. C) Violin plots of eccDNA size in the cancer cell lines investigated. The y-axis represents the log10 eccDNA size (bp) and the x-axis represents the cancer cell lines. [file mmc2.pdf]

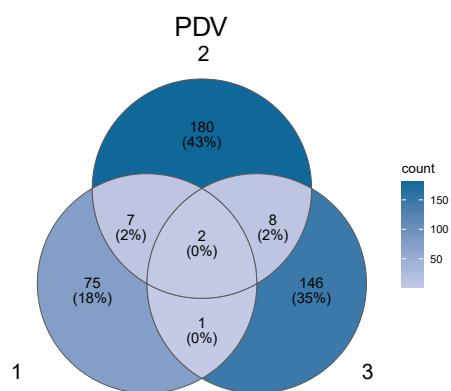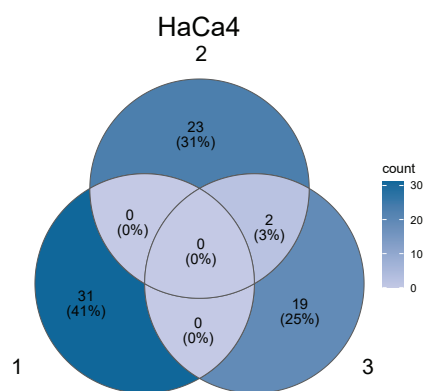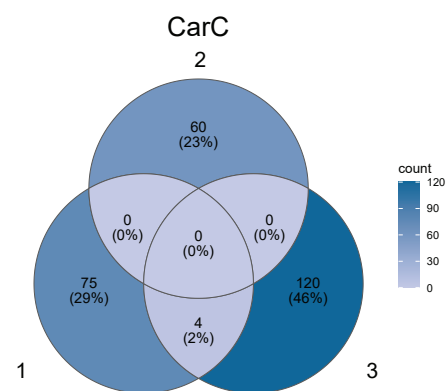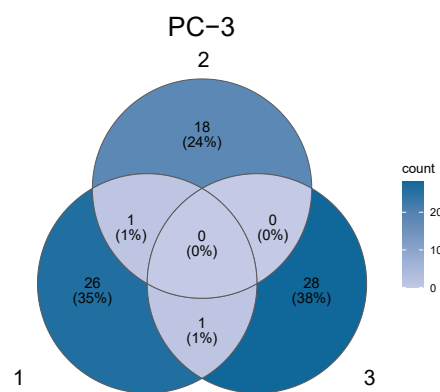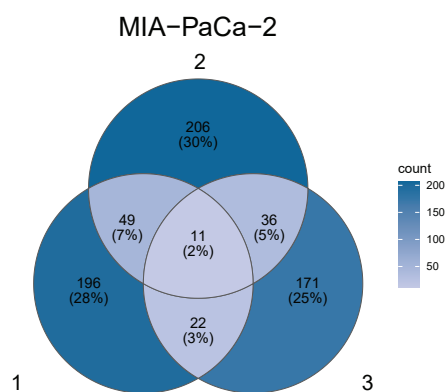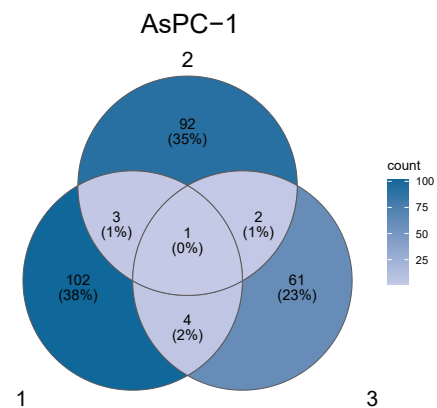

Supplement: Supplementary file 3 — Supplementary figure 3. Venn diagrams of unique full-length protein-coding genes located on eccDNA in the triplicates (designated 1, 2, and 3) of all the cell lines investigated. Full-length protein coding genes located on eccDNA found in three replicate of PDV: Olfr361, Ufsp1. Full-length protein coding genes located on eccDNA found in three replicate of MIA-PaCa-2: OR10J3, OR10A5, OR4L1, OR1E2, SCP2D1, KRTAP21–3, KRTAP19–8, H2BC10, ATP6V1G2, OR2A2, and ENSG00000287585. Full-length protein coding genes located on eccDNA found in three replicate of AsPC-1: TAF11L2. Of the total number of eccDNAs identified in each cell line between ∼ 0.1–0.2% carry a full-length protein coding gene. [file mmc3.pdf]

A

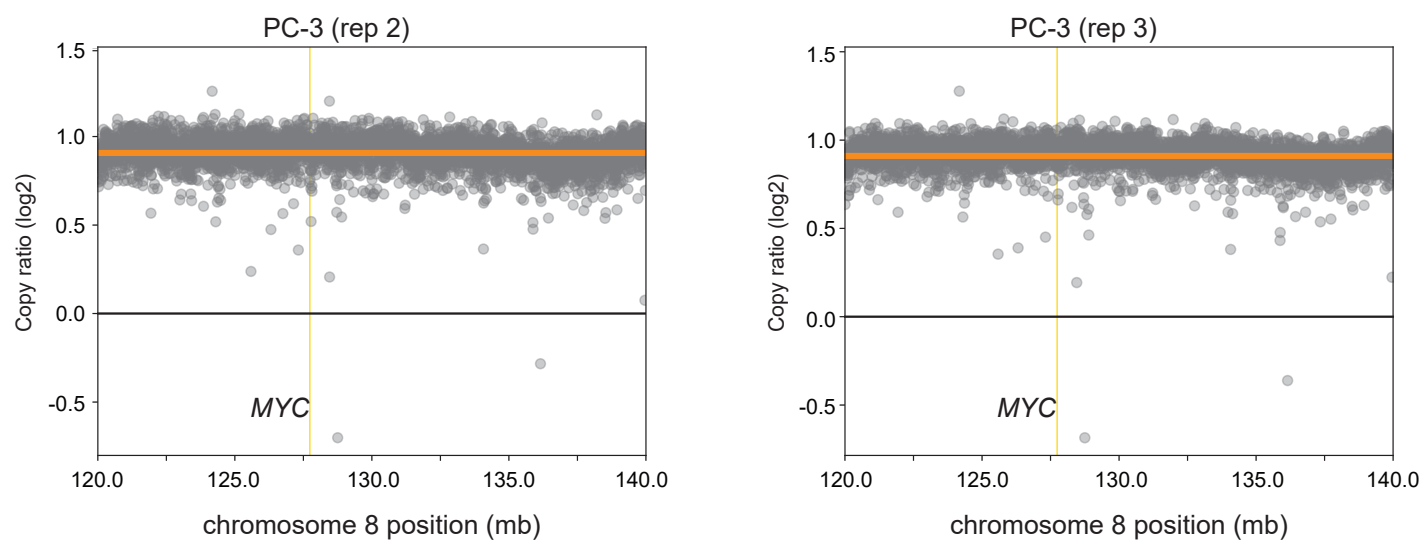

B

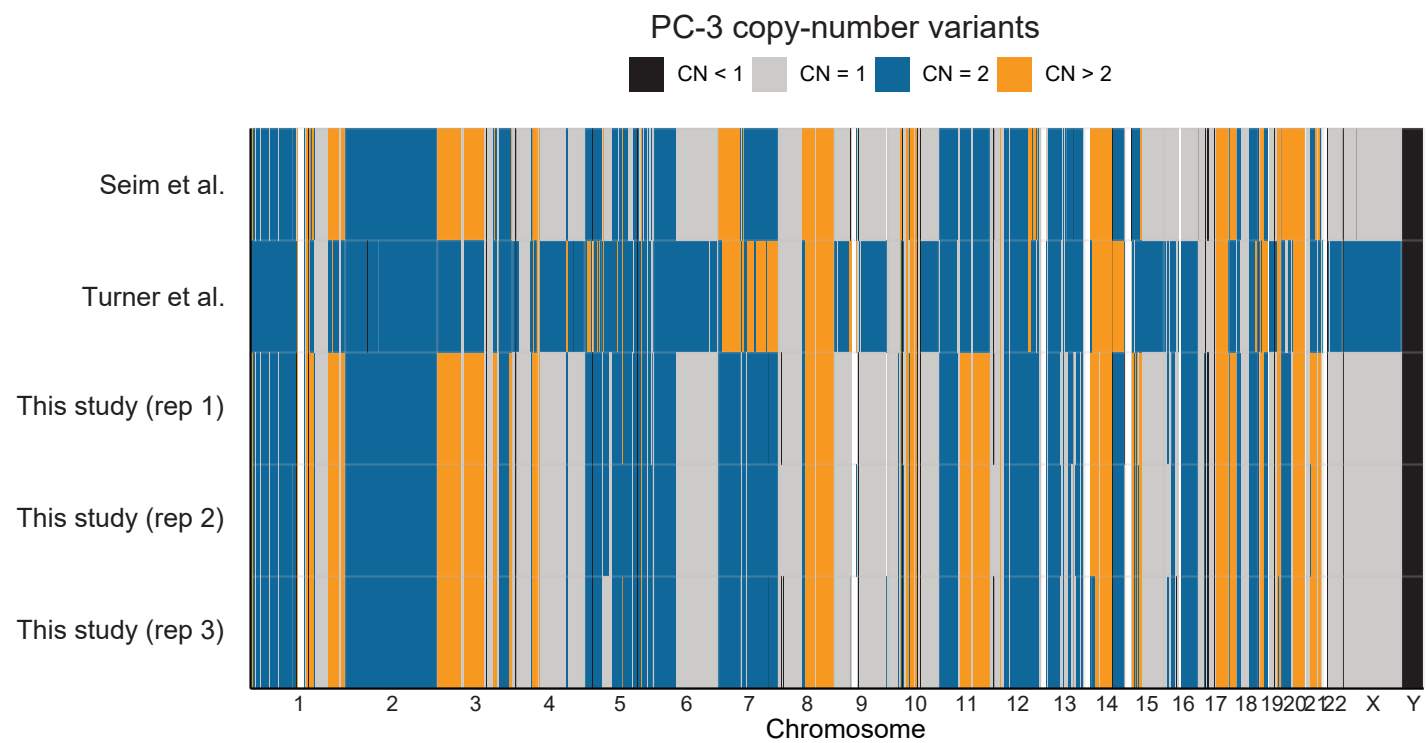

Supplement: Supplementary file 4 — Supplementary figure 4. Copy-number variations in PC-3. A) CNVkit scatterplot of chr8:120–140 Mb of the PC-3 replicate 2 and 3 from this study. B) Copy-number variations across all chromosomes calculated using CNVkit from the PC-3 triplicate WGS data, Seim et al. WGS data and Turner et al. WGS data. We note that the differences in copy number reported for the Turner et al. PC-3 isolate may be skewed as sequencing depth was low. [file mmc4.pdf]

A

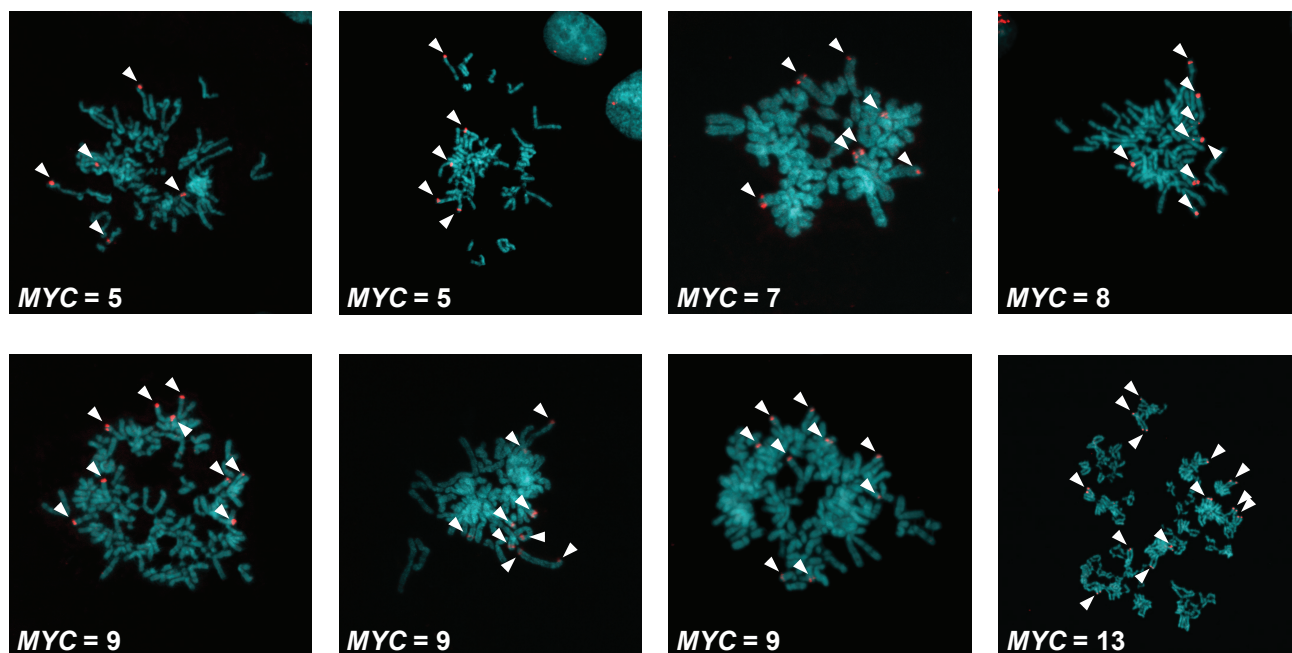

B

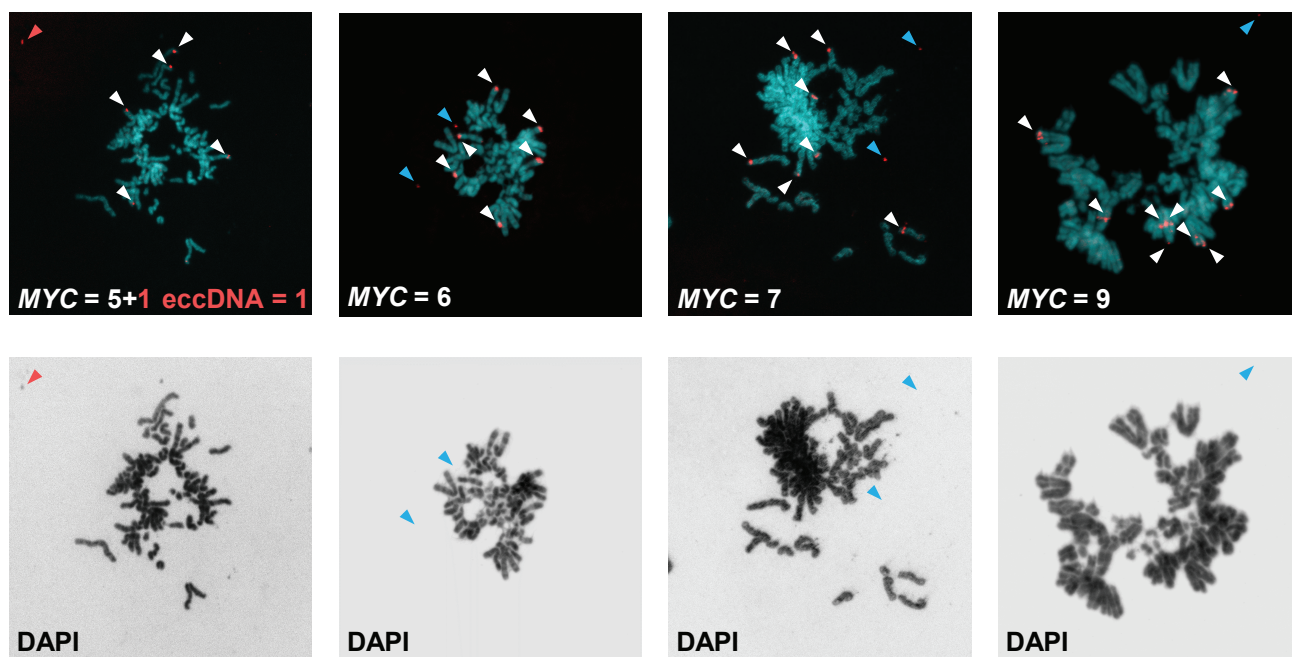

Supplement: Supplementary file 5 — Supplementary figure 5. FISH analysis PC-3 cells. A) FISH collection of full metaphase spreads with the MYC (red) specific probe and stained with DAPI for DNA detection (cyan). B) potential MYC carrying eccDNA candidates (eccDNA; red arrow) or unspecific signals (blue arrows). [file mmc5.pdf]
