# Supplementary material for: Variation of extrachromosomal circular DNA in cancer cell lines
Source: Comput Struct Biotechnol J. 2023 Aug 28;21:4207–14. doi: 10.1016/j.csbj.2023.08.027 (PMC10495552; doi:10.1016/j.csbj.2023.08.027)
Supplement: Supplementary file 6 — Supplementary material [file mmc6.docx]

Identified in three PC-3 replicates.

| Identified in replicate | Location | Genetic elements | ecDNA size (bp) | Estimated copy number |
| --- | --- | --- | --- | --- |
| 1, 2, 3 | ['chr10:33117036-37949668'] | ['ANKRD30A', 'CCNY', 'CREM', 'CUL2', 'FZD8', 'GJD4', 'MTRNR2L7', 'NRP1', 'PARD3', 'PARD3-AS1', 'PCAT5', 'ZNF248', 'ZNF33BP1'] | 4832632 | 13 |

Identified in two PC-3 replicates.

| Identified in replicate | Location | Genetic elements | ecDNA size (bp) | Estimated copy number |
| --- | --- | --- | --- | --- |
| 1, 2 | ['chr10:66716807-67000040'] | ['LRRTM3'] | 283233 | 7 |
| 2, 3 | ['chr14:90341382-90525404', 'chr14:90525994-90596034'] | ['CALM1'] | 254062 | 7 |

ecDNA identified in ours and Seim et al. PC-3 isolate.

| Identified in replicate/isolate | Location | Genetic elements | ecDNA size (bp) | Estimated copy number |
| --- | --- | --- | --- | --- |
| 1, 2, 3, Seim et al. | ['chr10:33117036-37949668'] | ['ANKRD30A', 'CCNY', 'CREM', 'CUL2', 'FZD8', 'GJD4', 'MTRNR2L7', 'NRP1', 'PARD3', 'PARD3-AS1', 'PCAT5', 'ZNF248', 'ZNF33BP1'] | 4832632 | 13 |
| 1, 2, Seim et al. | ['chr10:66716807-67000040'] | ['LRRTM3'] | 283233 | 7 |
| 1, Seim et al. | ['chr10:72748674-79332425', 'chr10:79333023-79532858', 'chr10:79940511-80227195'] | ['ADK', 'AGAP5', 'ANXA11', 'ANXA7', 'AP3M1', 'BMS1P4', 'BMS1P4-AGAP5', 'C10orf55', 'CAMK2G', 'CFAP70', 'CHCHD1', 'COMTD1', 'DLG5', 'DLG5-AS1', 'DNAJC9', 'DNAJC9-AS1', 'DUPD1', 'DUSP13', 'ECD', 'EIF5AL1', 'FAM149B1', 'FUT11', 'GLUD1P3', 'KAT6B', 'KCNMA1', 'KCNMA1-AS1', 'KCNMA1-AS2', 'KCNMA1-AS3', 'LRMDA', 'MRPS16', 'MSS51', 'MYOZ1', 'NDST2', 'NUDT13', 'OIT3', 'P4HA1', 'PLA2G12B', 'PLAC9', 'PLAU', 'POLR3A', 'PPIF', 'PPP3CB', 'PPP3CB-AS1', 'RPS24', 'SAMD8', 'SEC24C', 'SFTPD', 'SNORA11F', 'SNORD172', 'SYNPO2L', 'TMEM254', 'TMEM254-AS1', 'USP54', 'VCL', 'VDAC2', 'ZCCHC24', 'ZMIZ1', 'ZMIZ1-AS1', 'ZNF503', 'ZNF503-AS1', 'ZNF503-AS2', 'ZSWIM8', 'ZSWIM8-AS1'] | 7070270 | 14 |
| 1, Seim et al. | ['chr14:72666213-72684274', 'chr14:90341797-90453375', 'chr14:90454727-90459341', 'chr14:90489290-90525404', 'chr14:90525995-90595999', 'chr14:91108225-91160554'] | ['CALM1', 'SNORA11B'] | 292700 | 8 |
